# Isolation of enterococci, their antimicrobial susceptibility patterns and associated factors among patients attending at the University of Gondar Teaching Hospital

**DOI:** 10.1186/s12879-017-2363-3

**Published:** 2017-04-17

**Authors:** Amelework Yilema, Feleke Moges, Sisay Tadele, Mengistu Endris, Afework Kassu, Wondwossen Abebe, Getnet Ayalew

**Affiliations:** Ethiopia public health institute, P.O.Box-1242, Addis Ababa, Ethiopia

**Keywords:** Antimicrobial susceptibility, Associated factors, Enterococci

## Abstract

**Background:**

Enterococci become clinically important especially in immune compromised patients and important causes of nosocomial infections. Data on the prevalence, antimicrobial susceptibility patterns and associated factors of enterococci are scarce in Ethiopia.

**Methods:**

A hospital based cross-sectional study was conducted at the University of Gondar Teaching Hospital from February 28, 2014 to May 1, 2014. Pretested structured questionnaire was used to collect socio-demographic data and possible associated factors of enterococci infections. Clinical samples including urine, blood, wound swabs and other body fluids from patients requested by physician for culture and antimicrobial susceptibility test during the study period were included. A total of 385 patients were included in the study. Data were entered and analyzed using SPSS Version 20. *P* values <0.05 were considered as statistically significant.

**Result:**

The overall prevalence of enterococci infection was 6.2% (24/385). The commonest sites of infections were urinary tract followed by wound and blood. Among the 24 isolates, 33.3% (8/24) were resistant to all tested antimicrobial agents. Forty one point 7 % (10/24) of the enterococci isolates were vancomycin resistant enterococci (VRE). Moreover, two third of the isolates were multidrug resistant (MDR) enterococci. In multivariate analysis, duration of hospital stay for two days and more than two days with infection rate 17/32 (53.1%), previous history of any antibiotics (AOR = 9.13; [95% CI; 2.01–41.51] *P* = 0.00) and history of urinary catheterization (AOR = 8.80; [95% CI; 1.70–45.64] *P* = 0.01) were associated with presence of higher enterococci infections than their respective groups.

**Conclusion:**

The prevalence of enterococci infections among patients with UTIs, wound infections and sepsis were higher than the other infections. Multi drug resistant enterococci including VRE were isolated from clinical samples in the study area. Being hospitalized for ≥48 h, having history of any antibiotic administration and catheterization were associated factors for enterococci infections. Presence of VRE indicates decreased antibiotic treatment options of multidrug resistant enterococci. Therefore, efforts should be made to prevent enterococci infections and emergency of multidrug resistant enterococci. Moreover, species identification and antibiotic resistant in advanced and at large scale is demanding.

## Background

Enterococci are gram positive bacteria under family enterococcaceae which occur in pairs or short chains. Around 35 species have been identified and widely distributed in nature. They are normally found in the intestine, oral cavity, female genital tract of humans and animals [1]. Those organisms are catalase negative and facultative anaerobic organisms. Enterococci can able to proliferate in wide temperature range (5 °C-65 °C) and pH (4.5–10.0) [2]. In addition, they can grow in the presence of 6.5% NaCl. These characteristics differentiate them from streptococci. Moreover, Enterococci hydrolyze esculin in the presence of 40% bile [1].

A systematic review conducted on bacterial nosocomial infections showed that, enterococci were among 3^rd^ to 4^th^ leading cause of nosocomial infections worldwide. Among the isolates, multiple antimicrobial resistant enterococci were more prevalent including vancomycin resistant [3]. Studies indicate that enterococci are the second most common cause of urinary tract infections (UTI) and third most common cause of bacteremia from hospital acquired infections [4, 5].

According to the National Healthcare Safety Network summary report, between 2009 and 2010, enterococci were the second common cause of nosocomial infections. The report showed enterococci were 14%, next to *S aureus* (16%) and among these, 3% enterococci were vancomycin resistant [6].

A systematic review and meta-analysis had shown the prevalence of nosocomial infections in developing countries. The result showed enterococci were among the leading cause of nosocomial infections and they are next to *S aureus* and coagulase-negative Staphylococci. According to this review, high prevalence of enterococci were isolated from high risk patients, surgical site infections and blood stream infections [7]. Another systematic review on health care associated infections in Africa shows enterococci were next to *S aureus,* from gram positive bacteria [8].

Enterococci have naturally low level resistance to some antimicrobial agents and have high ability to acquire antibiotic resistant determinates. In spite of those multiple antimicrobial resistant enterococci are emerging as the leading cause of hospital acquired infections [5]. Especially, *E. faecalis* and *E. faecium* have become causes of international concern from this genus [9].

A study conducted in Gondar, Ethiopia indicated that 5.5% of the study participants had VRE; 7.8% of which were from HIV positive and 3.1% were from HIV negative study subjects [10].

Previous administration of antibiotic treatment, concurrent infections, surgery, catheterizations, duration of hospital stay, presence of pervious hospitalization and underlying diseases like cancer, HIV and diabetics are among the risk factors associated with the spread of enterococci infection [3, 11]. Enterococci cause infections mainly in immune compromised patients. However, increasing number of immune compromised individuals, because of different reasons, increases the spread and risk of enterococci infections [5].

Prevalence, antimicrobial susceptibility patterns and associated factors of enterococci infections were reported in some countries of America, Europe, Asia and Africa. In spite of this organism being among the leading cause of hospital acquired infection in the world, documented data about the prevalence, their antimicrobial susceptibility patterns and associated factors of enterococci infections are scarce in this study area. Therefore, this study was conducted to determine the prevalence, their antimicrobial susceptibility patterns and associated factors of enterococci among patients attending at the University of Gondar Teaching Hospital (UoGTH).

## Method

### Study design and period

Hospital based cross-sectional study was conducted from February 28, 2014 to May 1, 2014. The study was conducted at the UoGTH. University of Gondar Teaching Hospital is a tertiary level teaching hospital that provides service to over five million inhabitants in North West Ethiopia, and located 737 Km to the North from the capital city, Addis Ababa. The hospital consists of different units including intensive care unit (ICU) with 18 beds, 13 wards with 510 beds, outpatient departments and the diagnostic laboratory. The diagnostic laboratory divided into different sections. Culture and antimicrobial susceptibility testing is among the services in microbiology section of the laboratory.

### Study population

The study populations were patients who have been requested by physicians for culture and antimicrobial susceptibility test during the study period. Cognizant of the fact those enterococci are normal flora of some specific sites like respiratory, genital and gastro-intestinal tract. In spite of this, samples such as; sputum, throat swab, stool, and vaginal swabs were excluded from the study.

### Sample size determination

Since there is no previous study conducted in this area, 50% prevalence (anticipated proportion) was used for sample size determination by using a single population proportion formula. A total of 385 patients were included for this study. Systematic random sampling was performed to select the study participants. A review of two years laboratory record prior to the study period revealed that average 13 clinical samples (i.e. urine, blood, swabs and other body fluids) have been recruited for culture and antimicrobial susceptibility, per day. Accordingly, the total participants to be included in this study in 2 months period were calculated to be 780. Therefore, the sampling interval was calculated as N/n; N = the total estimated number of samples in two month, n = required sample size. Hence, the sampling interval has been drawn to be 2.

### Data collection

Socio-demographics and other independent variables were collected from each study participants by using self-structured questionnaire. Data collection was done by principal investigator under the supervision of the advisors. The data was collected from their places of the study participant’s from outpatient departments or inpatient wards.

### Sample collection and transportation

Clinical samples were collected from each study participant aseptically. For adult study participants, 5 to 10 ml of blood samples were collected using two blood culture bottles per participant, prepared in our laboratory. On the other hand, 2.5-10 ml and 0.5-5 ml blood samples were collected for pediatrics and infants, respectively. Five to 10 ml of morning mid-stream urine samples were collected and processed within 2 h of collection. At least 1 ml of Cerebrospinal fluid (CSF) and other body fluids were collected aseptically. Swab of wound secretion, pus, purulent exudates or wound discharge were aseptically obtained using sterile cotton swab [12, 13]. Each collected samples were transported to the bacteriology laboratory in the biomedical complex at the School of Biomedical and Laboratory Sciences. All swabs were transported with Brain Heart Infusion (BHI) broth.All the process of sample management has been conducted according to a pre-established standard operating procedure.

### Bacterial isolation and identification

The blood culture bottles were incubated at 37 °C and were observed daily after 48 h for 5 consecutive days for presence of turbidity, hemolysis, gas formation or color changes which are evidence of microbial growth. If the culture bottle does not show any growth within 7 days, it was reported as negative. Whenever visible growth appears, the bottle was opened aseptically; a small amount of broth was taken with a sterile loop and sub cultured on Bile-esculina zide agar (BEAA) (Hardy Diagnostics, Santa Maria, CA, United states) [12, 14].

Urine samples were inoculated on BEAA media with a 10 μl calibrated loop and incubated at 37 °C for 24 h [14]. Presence of 10^4^ colony forming unit per ml of bacteria with black colored colony was considered as significant enterococci in the urine. Other clinical samples were directly inculcated on BEAA at 37 °C for 24 h and checked for growth of black colored colony [13, 14]. The presence of enterococci were confirmed by further tests such as gram stain, catalase reaction, growth on broth containing 6.5% NaCl and growth in BHI broth, at 37 °C and 45 °C for 48 h, respectively [14, 15].

### Antimicrobial susceptibility testing

Antimicrobial susceptibility testing was done using Kirby-Bauer disk diffusion method on Muller Hinton agar based on Clinical Laboratory Standards Institute (CLIS), 2012. Isolated enterococci were tested against ampicillin (10 μg), penicillin (10 IU), vancomycin (30 μg), erythromycin (15 μg), chloramphenicol (30 μg), doxycycline (30 μg), amoxicillin/clavulanic acid (20/10 μg), ciprofloxacin (5 μg) and tetracycline (30 μg) [16] and all are Oxoid (Basingstoke, Hampshire, England).

### Quality control

All culture media were prepared following the manufacturer’s instruction. Batch of prepared media was checked for sterility by incubating samples of the plate at 37 °C for 24 h. Moreover, *E. faecalis* ATCC 29212, *E. coli* ATCC 25922, *S. pyogenes* ATCC 19615 and *S. aureus* ATCC 25923 standard strains were used as a quality control [12, 16].

### Data analysis

Data were entered and analyzed using SPSS Version 20. Prevalence of enterococci was determined using proportion of population under the study. Frequency distributions of the socio-demographic characteristics were described. The bivariate analysis using maximum likelihood estimates of the categorical variables was done using a cross tabulation to determine the association of the variables. Odds ratio was calculated with their respective confidence interval. Furthermore, the variables with *P* < 0.05 in the bivariate analysis were subjected to conditional logistic regression to get the independent predictors of enterococci infections by adjusted odds ratio (AOR) together with their respective confidence interval.

## Results

### Prevalence of enterococci

Out of the 385 study participants, the prevalence of enterococci infection in this study was found to be 6.2% (24/385). Of which, in-patients and out-patients accounted for 6.8% (20/24) and 4.4% (4/24), respectively of the study participants (Table 1).

**Table 1:** Socio-demographic characteristics of the study participants and prevalence of enterococci infection at the UoGTH, February 28, 2014 to May 1, 2014.

Socio-demographics	Frequency (%)	Enterococci infection	
		Present no. (%)	Absent no. (%)
Sex			
Female	176 (45.7)	10 (5.7)	166 (94.3)
Male	209 (54.3)	14 (6.7)	195 (93.3)
Age (years)			
<5	123 (31.9)	12 (9.8)	111 (90.2)
5–14	36 (9.4)	1 (2.8)	35 (97.2)
15–30	108 (28)	3 (2.8)	105 (97.2)
31–50	83 (21.6)	3 (3.6)	80 (96.4)
>50	35 (9.1)	5 (14.3)	30 (85.7)
Residence			
Rural	169 (44.0)	13 (7.7)	156 (92.3)
Urban	216 (56.0)	11 (5.1)	205 (94.9)
Patient setting			
Out-patient	92 (23.9)		88 (95.6)
In-patient	293 (76.1)	20 (6.8)	273 (93.2)
Educational status			
Illiterate	180 (46.8)	11 (6.1)	169 (93.9)
Literate	205 (53.2)	13 (6.3)	192 (93.7)
Total	385 (100.0)	24 (6.2)	361 (93.8)

### Socio-demographic characteristics of the study participants

Fifty four point 3 % (209/385) and 45.7% (176/385) of the study participants were males and females, respectively. The median age of the study participants was 20 years old with inter quartile range of 2–36 years. Majorities, 76.1% (293/385) of the participants were in-patients and 23.9% (92/385) were out-patients (Table 1).

Majority of the isolate were recovered from urine, wound and blood, 10/24 (41.6%), 6/24 (25.0%), and 5/24 (20.8%), respectively (Table 2). There were 71 urines, 44 wounds, 47 bloods, 14 ascetics, 9 ear discharges, 4 abdominal abscess, 111 CSFs, 23 peritoneal fluids, 51 Plural fluids, 7 eye discharges and 4 synovial fluids clinical samples were analyzed.

**Table 2:** Distribution of enterococci in different clinical samples (*n* = 24) at the UoGTH, February 28, 2014 to May 1, 2014

Type of sample	Frequency (%)
Abdominal abscess	1 (4.2)
Ascetic	1 (4.2)
Blood	5 (20.8)
Ear discharge	1 (4.2)
Urine	10 (41.6)
Wound	6 (25.0)
Total	24 (100.0)

### Antimicrobial susceptibility patterns

Among the 24 isolates from different clinical samples, 41.7% (10/24) were resistant to vancomycin (Table 3). Of the 10 VRE, 3 were community acquired.

**Table 3:** Antimicrobial susceptibility patterns of enterococci (*n* = 24) at the UoGTH, February 28, 2014 to May 1, 2014

Antimicrobial discs	Susceptible no. (%)	Resistant count (%)
Amoxicillin/clavuanic acid	8/24 (33.3)	16/24 (66.7)
Ampicillin	8/24 (33.3)	16/24 (66.7)
Chloramphenicol	7/24 (29.2)	17/24 (70.8)
Ciprofloxacin	7/24 (29.2)	17/24 (70.8)
Doxycycline	6/19 (32)	13/19 (68)
Erythromycin	5/14 (36)	9/14 (64)
Penicillin	8/24 (33.3)	16/24 (66.7)
Tetracycline	6/19 (32)	13/19 (68)
Vancomycin	14/24 (58.3)	1024 (41.7)

Multidrug resistances (MDR) were observed in 75% (18/24) of enterococci isolates and isolates resistant to all antimicrobials tested were 33.3% (8/24). All the isolated VRE were MDR (Table 4).

**Table 4:** Profile of multidrug resistant enterococci isolates at the UoGTH, February 28, 2014 to May 1, 2014

Resistance rate	Combination of antibiotics	No. (%) of isolates tested
R5	TE, E, DOXY, CIP, CAF	1
R5	TE, E, DOXY, CIP, VAN	1
R6	E, CIP, CAF, AMP, P, AMC	1
R7	TE, E, CIP, CAF, AMP, P, AMC	1
R8	TE, E, DOXY, CIP, CAF, AMP, P, AMC	5
R8	TE, E, DOXY, CAF, AMP, P, AMC, VAN	1
R9	TE, E, DOXY, CIP, CAF, AMP, P, AMC, VAN	8
Total		18

Key; *E* erythromycin, *TE* tetracycline, *AMP* ampicillin, *P* penicillin, *AMC* amoxicillin/clavuanic acid, *CAF* chloramphenicol, *CIP* ciprofloxacin, *DOXY* doxycycline, *VAN* Vancomycin, *R* Resistance.*R2-R9* Number of antibiotics resistance from 2 to 9, respectively. *MDR* organisms resistant to >2 antibiotics

### Factors associated with enterococci infections

All socio-demographic characteristics were not significantly associated with enterococci infections. However, from clinical factors such as, duration of hospital stay, history of hospitalization, previous usage of any antibiotics, urinary catheterization, and laboratory confirmed bacterial infection were statistically significant (*P* value <0.05) by binary analysis (Table 5).

**Table 5:** Bivariate analysis of enterococci infections at the UoGTH, February 28, 2014 to May 1, 2014

Characteristics	Total No. (%)	Enterococci infection		COR (95% CI)	*P*- value
		Presence No. (%)	Absence No. (%)		
Total	385 (100%)	24 (6.2%)	361 (93.8%)		
Sex					
Female	176	10 (5.7)	166 (94.3)	1.19 (0.51–2.75)	0.68
Male	209	14 (6.7)	195 (93.3)	1.00	
Age					0.06
<5	123	12 (9.8)	111 (90.2)	0.26 (0.03–2.10)	0.21
5–14	36	1 (2.8)	35 (97.2)	0.26 (0.07–0.96)	0.04
15–30	108	3 (2.8)	105 (97.2)	0.35 (0.10–1.27)	0.11
31–50	83	3 (3.6)	80 (96.4)	1.54 (0.50–4.72)	0.45
>50	35	5 (14.3)	30 (85.7)	1.00	
Hospital stay (hours)					
<48	353	7 (2)	346 (98)	1.00	
≥48	32	17 (53)	15 (47)	56.02 (18.32–178.39)	0.00
HIV					
Negative	367	22 (6)	345 (94)	1.00	
Positive	18	2 (0.1)	16 (99.9)	1.96 (0.00–9.79)	0.39
CD4 count^a^					
<200	6	0 (0.0)	6 (100)	0.00 (0.00–9.76)	
>200	12	2 (16.7)	10 (83.3)	1.00	0.52
HAART^a^					
Pre	9	2 (22.2)	7 (87.8)	1.00	
On	9	0 (0.0)	9 (100)	0.00 (0.00–4.38)	0.47
WHO stage^a^					1.00
I	5	2 (40)	3 (60)	1.00	
II	1	0 (0.0)	1 (100)	0.00 (0.00–60.98)	1.00
III	6	0 (0.0)	6 (100)	0.00 (0.00–3.71)	0.18
IV	6	0 (0.0)	6 (100)	0.00 (0.00–3.71)	0.18
History of:					
Animal contact	111	10 (9)	101 (90)	1.84 (0.73–4.58)	0.15
Antibiotics usage	54	14 (25.9)	40 (74.1)	11.24 (4.34–29.47)	0.00
Cancer	3	1 (33.3)	2 (66.7)	7.80 (0.00–116.25)	0.18
Catheterization	17	11 (64.7)	6 (35.3)	50.06 (14.24–184.31)	0.00
Diabetics	9	0 (0.0)	9 (100)	0.00 (0.00–9.28)	1.00
Cotrimoxazole prophylaxis^a^	36	2 (5.6)	34 (94.4)	0.87 (0.20 - 3.88)	1.00
Hospitalization	40	11 (27.5)	29 (72.5)	9.69 (3.66–25.68)	0.00
Immunosuppressive usage	51	3 (5.9)	48 (94.1)	0.93 (0.21–3.47)	1.00
Laboratory confirmed Bacterial infection	10	3 (30)	7 (70)	7.22 (1.36–34.43)	0.02
Prosthetic device usage	10	1 (10)	9 (90)	1.70 (0.21–14.01)	0.48
Surgery	6	0 (0.0)	6 (100)	0.00 (0.00–14.88)	1.00
UTI	55	2 (3.6)	53 (96.4)	0.53 (0.08–2.42)	0.55

^a^Only for HIV positives

In the multivariate analysis, presence of enterococci infections were significantly associated with ≥48 h hospital stay with infection rate: 17/32 (53.1%), history of urinary catheterization (AOR = 8.80; [95% C.I, 1.70–45.64]; *P* = 0.01) and previous administration of any antibiotics (AOR = 9.13; [95% C.I, 2.01–41.51]; *P* = 0.00). History of hospitalization and laboratory confirmed bacterial infection were not associated (Table 6).

**Table 6:** Multivariable analysis of enterococci infections at the UoGTH, February 28, 2014 to May 1, 2014

Factors	AOR (95% C.I)	*P*-value
Age		
<5	1.67 (0.22–12.56)	0.62
5–14	3.51 (0.20–60.29)	0.39
15–30	0.66 (0.07–6.04)	0.71
31–50	0.33 (0.34–3.08)	0.33
>50	1.00	
Hospital stay ≥48 h	63.31 (16.09–249.07)	0.00
History of:		
Animal contact	1.80 (0.33–9.808)	0.50
Antibiotic usage	9.13 (2.01–41.51)	0.00
Cancer	0.26 (0.01–9.73)	0.47
Urinary catheterization	8.80 (1.70–45.64)	0.01
Laboratory confirmed bacterial infection	0.24 (0.01–5.79)	0.38
Hospitalization	2.10 (0.26–16.57)	0.49

## Discussion

In this study, the overall prevalence of enterococci was found to be 6.2%. This was in line with report from Nigeria 5.9% [17]. However, it was lower than from annual summary reported to center for diseases control and prevention which was 14% [6] and also report from Saudi Arabia 31.71% [18]. The lower prevalence in the present study might be due to the variation in the study participants and the methods employed for detection of enterococci. That is, the study subjects included in the previous studies were hospitalized patients as their aim was to show hospital acquired infections. Moreover, the method used in Saudi Arabia was molecular technique that has a better sensitivity and hence higher prevalence rate has been reported.

On the other hand, the prevalence in the present study was higher than report from Kenya 0.22% [19]. This variation might be because of the Kenyan study participants included only outpatients. In addition, studies conducted in Ethiopia at deferent hospitals including Jimma, Felege Hiwot and UoGTH reported lower prevalence of enterococci which accounted for 0.59%, 0.64% and 2.13%, respectively [20–22]. The variation might be explained by the use of enterococci selective media in the current study which was not used in all the other studies. Moreover, the gradual increase in the prevalence of enterococci infections might have contributed to the increased prevalence as evidenced by other studies [6].

Majority of the enterococci isolates were detected from three clinical specimens: urine, wound discharge and blood with the recovery rate of 41.6%, 25.0% and 20.8%, respectively. This result was consistent with a previous report from India which reported enterococci associated UTI, wound infection, and sepsis of 39.3%, 15.3% and 2% respectively [9]. However, this result showed different patterns from a study conducted in Saudi Arabia; UTI 55%, sepsis 16% and wound infection 9% [18] and another study in India; UTI 17.2%, bacteremia 8.3%, and wound infection 1.7% [23]. In general, this showed that entrococci are common causes of UTI, wound infection and bacteremia.

In this study, vancomycin had better antimicrobial susceptibility to enterococci than the rest of antibiotics including gampicilin, penicillin, amoxicillin/clavuanic acid, ciprofloxacin and chloramphenicol. This result was similar with study conducted in Egypt with the susceptibility of vancomycin then to ampicilin, ciprofloxacin and chloramphenicol [24].

Most of the isolates were resistant to the tested antibiotics erythromycin 64%, ampicilin, penicillin and amoxicillin/clavuanic acid 66.7% each, doxycycline 68%, tetracycline 68%, ciprofloxacin 70.8% and chloramphenicol 70.8%. The resistance patterns observed in the current study were higher than the previous study in Gondar, except for penicillin where a similar resistance pattern was recorded [25]. This variation might be the gradual change in antibiotic resistance pattern. However, the resistance patterns observed in this study was less than another previous study done in Gondar, Ethiopia against ampicilin 100% and erythromycin 87.5% [10]. In the case of the previous study, the participants were all HIV patients who use different antibiotics along highly active antiretroviral therapy that might be contributed for emergence of high rate of drug resistance microorganisms including enterococci compared to other patients [26].

Of the 24 isolated enterococci, 41.7% were resistant to vancomycin. This result was in line with a finding in Nigeria 42.9% [17]; but lower than in Iraq 71.4% [27] and Serbia 54.05% [28]. This discrepancy might be due to the difference in type of clinical samples analyzed as it was on urine in the case of Iraq and blood in case of Serbia. The difference in the overall antibiotic resistance in the population might have also its own contribution. The current result is also higher than a finding from Egypt 9.00% [24]. This variation might be due to a gradual increase in the emergence of VRE as indicated in other reports [6] and the difference in the overall antibiotic resistance in the population.

Of the 10 VRE, 3 were isolated from out-patients, in this study. Similarly, 3.1% community acquired VRE were also reported from the same hospital from the stool samples of blood donors [10]. These shows VRE are circulating in the community and might be disseminated to the environment. Vancomycin resistance genes are easily transmitted though transposons which contain van genes and enterococci have high ability to harbor and transfer transposable genetic elements [9]. Even these genes now a days are disseminated to other pathogenic organisms and recently vancomycin resistant *S.aureus* were also reported [9, 28].

Multiple drug resistant enterococci were observed among 75% of the isolates. However, this finding was higher than the previous study held in Gondar, Ethiopia, reported as 33.33% [25]. This discrepancy might be due to the gradual change in the MDR strains due to the selective pressure. The result also lower compared to study in Iraq where all the isolates were MDR 100% [29]. This variation might be due to difference in the study participants and type of specimen which was only hospitalized patients with UTI in the previous study. This study also revealed that, 33.3% of enterococci isolates developed resistance to all tested antibiotics which was almost similar with report from Nigeria 42.86% [28].

Enterococci infections have been reported as high among medical device mediated hospitalized population. In this study, patients with a recent history of urinary catheterization were at 9 fold with [95% C.I 1.70–45.64] risk of getting enterococci infections as compared to those with their respective groups. This result was similar with study conducted in Spain [30]. The reason for the higher prevalence in patients waited in the hospital for longer period might be due to the increase chance of insertion of different medical equipment’s including catheter which are some of the major routes for transmission of nosocomial enterococci infection in many hospitals [3, 5].

This study showed high prevalence of enterococci infection with patients having history of previous administration of antibiotics 25.9% compared to no history of previous administration of any antibiotics 3.0% and the difference was statistically significant (AOR = 9.13; [95% C.I, 2.01–41.51]). The association of enterococci infections and history of antibiotic exposure had similar result with study conducted in Germany (AOR = 3.88; [95% CI; 1.18–12.63]) [31].

Enterococci infections have been reported, being inpatient two days and more than two days hospital stay significantly associated with enterococci infections compared with their respective groups and the prevalence were found to be 53.1% and 1.98%, respectively. The reason for the survival of enterococci in hospital environment might be explained by their intrinsic resistance to several commonly used antibiotics and their ability to acquire resistance genes. In addition, the selective pressure of the sensitive strains due to antibiotic usage has a great role for the increment of antibiotic resistant enterococci [5]. Moreover, mostly hospitalized patients stay for longer period are immunocompromised because of different reasons such as chronic disease like diabetics, cancer, HIV, etc. and enterococci infections are high in those patients [3].

## Limitations of the study

Species identification was not investigated due to resource shortage and the study period was short.

## Conclusions

The prevalence of enterococci infection among patients with UTIs, wound infections and sepsis were higher than the other patients attending health facilities. Multidrug resistant enterococcus was found to be 75%. Vancomycin resistance enterococcus was also significantly higher among the study population. Being inpatient two days and more than two days, having history of any antibiotic administration or urinary catheterization were associated factors for enterococci infection.

Patients attending health facilities for the cases of UTIs, wound infections and sepsis have to be critically examined for enterococci infection. Evidence on antimicrobial susceptibility testing of enterococci infections should be available before prescription of antibiotics and promoting rational drug use. Attention has to pay for inpatient, patients having history of any antibiotics or urinary catheterization for the suspension of enterococci infections. Finally, further study on species identification and antibiotic resistant in advanced and at large scale is demanding.

## Abbreviation

AOR: Adjusted odds ratio
ATCC: American type culture collection
BEAA: Bile-EsculinAzide Agar
BHI: Brain heart infusion
CI: Confidence interval
CLIS: Clinical laboratory standards institute
CSF: Cerebrospinal fluid
HIV: Human immunodeficiency virus
ICU: Intensive care unit
MDR: Multidrug resistant
NaCl: Sodium chloride
NICU: Neonatal intensive care unit
UTI: Urinary Tract Infections
UoGTH: University of Gondar Teaching Hospital
VRE: Vancomycin resistant enterococci
